# The predictive value of red cell distribution width for stroke severity and outcome

**DOI:** 10.1186/s13104-020-05125-y

**Published:** 2020-06-15

**Authors:** Kavous Shahsavarinia, Younes Ghavam Laleh, Payman Moharramzadeh, Mahboob Pouraghaei, Elyar Sadeghi-Hokmabadi, Fatemeh Seifar, Farid Hajibonabi, Zhila Khamnian, Mehdi Farhoudi, Sara Mafi

**Affiliations:** 1 Neuroscience Research Center, Tabriz University of Medical Sciences, Tabriz, Iran; 2 Emergency Medicine Research Team, Tabriz University of Medical Sciences, Tabriz, Iran; 3 Stem Cell Research Center, Aging Research Institute, Faculty of Medicine, Tabriz University of Medical Sciences, Tabriz, 5166/15731 Iran; 4 Student Research Committee, Tabriz University of Medical Sciences, Tabriz, Iran; 5 Department of Community and Family Medicine, Tabriz University of Medical Sciences, Tabriz, Iran

**Keywords:** Ischemic Stroke, Tissue Plasminogen Activator, Red Cell Distribution Width, Modified Ranking Score, National Institute of Health Stroke Scale, Prediction

## Abstract

**Objectives:**

In the present study, we sought to investigate the association between red cell distribution width (RDW) and stroke severity and outcome in patients who underwent anti-thrombolytic therapy with tissue plasminogen activator (tPA).

**Results:**

In this prospective study, 282 stroke patients who underwent tPA injection were included. The categorization of RDW to < 12.9% and > 13% values revealed insignificant difference in stroke severity score, accounting for the mean 36-h NIHSS of 8.19 ± 8.2 in normal RDW values and 9.94 ± 8.28in higher RDW group (p = 0.64). In seventh day, NIHSS was 6.46 ± 7.28 in normal RDW group and was 8.52 ± 8.35 in increased RDW group (p = 0.058). Neither the 36-h, nor the seventh day and 3-month mRS demonstrated significant difference between those with normal and higher RDW values.

## Introduction

Cerebral ischemic attack is a general term used for ischemic stroke, including cerebral thrombosis, embolism, and lacunar infarction. Seventy percent of cerebrovascular attacks are related to ischemic stroke, which is induced by a disorder in the brain–blood supply lesions. Ischemic stroke is the second most common cause of death, accounting for more than 20 million disabilities worldwide [1, 2]. However, tissue plasminogen activator (tPA) antithrombotic therapy, a recently introduced treatment for ischemic stroke, has had promising results [3, 4].

A predictive parameter for stroke severity would enhance the antithrombotic therapeutic approach, which may reduce the high incidence and mortality rate of ischemic stroke [5, 6]. Red cell distribution width (RDW) is a hematologic parameter that indicates the divergence of red blood cell volume. Increases in RDW have been found in different physiological and pathological conditions such as pregnancy, vitamin B12 and folate deficiency, malignancy, idiopathic pulmonary fibrosis and coronary artery disease [7–12]. Although RDW was created for the diagnosis of different types of anemia, recent research has revealed its predictive role in cerebrovascular diseases [13].

Different studies have shown an increased value of RDW after ischemic stroke [14–18]. Feng et al. suggested that increased inflammation and oxidative stress during ischemia result in elevation in RDW and are associated with poor prognosis. However, it is still unclear that if RDW can predict the treatment response in stroke patients who are receiving antithrombotic therapy [16]. Therefore, the present study aimed to investigate RDW’s ability to predict stroke severity and antithrombotic treatment outcomes in ischemic stroke patients. We hypothesized that RDW is a predictive marker for treatment response in patients receiving antithrombotic therapy.

## Main text

### Materials and methods

This prospective study was conducted over 18 months, starting in April 2016. The participants were patients with definitive stroke diagnoses with specific criteria for tPA injection [19] who were admitted to the emergency department of Imam Reza University Hospital, Tabriz University of Medical Sciences. Those with transient ischemic attacks, intracerebral hemorrhage, cerebral sinus venous thrombosis, subarachnoid hemorrhage, renal insufficiency and pregnant women were excluded from the study population.

Blood samples were obtained at the time of admission to measure RDW. The patients completed follow-up examinations after 3 months. The severity of the stroke was assessed using the National Institute of Health Stroke Scale (NIHSS), and the clinical outcome was measured using the Modified Rankin Score (mRS) at 36 h, 7 days, and 3 months.

Blood samples were mixed with Ethylenediaminetetraacetic acid (EDTA) and RDW was calculated within 3 h of admission using the Sysmex KX-21 automated cell counter (Sysmex Corporation, Kobe, Japan). blood samples. RDW values of ≤ 12.9 were categorized as normal and values ≥ 13.0 were considered elevated. Stroke severity (NIHSS) was categorized as mild (0–6), moderate (7–15), and severe (16–38), and mRS ≪ 2 (0,1, and 2) was defined as a fine outcome.

Statistical analysis was performed using SPSS software version 19.0 (IBM Corp., Armonk, NY, USA). The categorical variables were calculated as percentages, and the continuous variables were calculated as mean ± standard deviation. The continuous variables were compared using independent t-tests (for normally distributed data [20]), and the categorical variables were compared using qui-square tests. The receiver operating characteristics (ROC) was performed to ensure the accuracy of RDW in detection of stroke severity, where an area under curve (AUC) close to 1 was considered to be a test with high predictive value. A multivariant linear regression analysis was conducted for correction of any other confounding factor (age, gender, hypertension, diabetes, hyperlipidemia, smoking, time of admission). A *P* value < 0.05 was regarded as significant.

## Results

### General findings

Two-hundred eighty-two patients including 155 men and 127 women, aged 65.20 ± 12.70 years (17–90) were enrolled.

The mean NIHSS score was 13.16 ± 6.39 at time of admission, and it took averagely 55.99 ± 30.12 h for tPA injection (5–184 h). The NIHSS score improved significantly after the injection in 36 h (p = 0.027) with the mean 36-h score of 10.10 ± 8.93.

RDW values ranged from 10.4% to 20.5%, with the mean value of 13.67 ± 1.17%.

Mean values for RDW did not significantly correlate with the severity of stroke (p = 0.11). In mild form of stroke (NIHSS = 0–6), the mean value for RDW was 13.60 ± 0.22% and in stroke of moderate severity (NIHSS = 7–15), it was 13.58 ± 0.11%. For patients with severe stroke (NIHSS > 16), the mean RDW was higher than the mild to moderate cases with mean value of 13.854 ± 0.12%.

Table 1 demonstrates patients’ characteristics according to the severity of stroke.

**Table 1:** Patients’ characteristics based on the severity of stroke

	Mild (N = 43)	Moderate (N = 134)	Severe (N = 105)
Age (years)	59.72 ± 10.68	65.46 ± 13.185	67.01 ± 12.38
Gender			
Female	18	48	61
Male	25	86	44
Medical condition			
Hypertension	56.41%	62.59%	66.01%
Diabetes	23.07%	20.61%	27.18%
Hyperlipidemia	30.76%	22.90%	26.21%
Smoking current smoker	30.76%	25.95%	17.47%
Past history of smoking	38.46%	26.71%	19.41%
History of past ischemic attacks	2.56%	9.92%	12.62%
Congestive heart failure	10.25%	6.10%	12.62%
Arrhythmia	2.56%	9.16%	22.33%
Ischemic heart disease	15.38%	19.84%	22.33%
Imaging modalities			
Ejection fraction	48.8 ± 1.8	50.36 ± 0.96	48.47 ± 1.25
Pathologic CT scan finding	0%	2.1%	10.38%
Laboratory tests			
White blood cell count	7724.00 ± 444.1	7828.57 ± 296.5	9110.20 ± 900.7
Hemoglobin (mg/dl)	13.50 ± 0.46	14.15 ± 0.22	13.32 ± 0.28
Hematocrit (percent)	39.83 ± 1.01	42.09 ± 0.57	40.32 ± 0.70
Platelet	246200 ± 19363	246714.29 ± 10291.12	239469.39 ± 10080.33
RDW (percent)	13.78 ± 0.30	13.59 ± 0.15	12.40 ± 0.56
PDW (percent)	12.72 ± 0.32	12.50 ± 0.29	12.70 ± 0.29
PT	13.16 ± 0.14	13.22 ± 0.10	13.53 ± 0.13
PTT	28.0 ± 0.40	27.75 ± 0.34	29.57 ± 0.98
INR	1.03 ± 0.01	1.07 ± 0.01	1.11 ± 0.01
Blood glucose (mg/dl)	159.08 ± 17.83	134.26 ± 6.32	8481.29 ± 6369.74
Creatinine (mg/dl)	1.03 ± 0.04	1.00 ± 0.02	1.06 ± 0.10
Total cholesterol (mg/dl)	174.00 ± 8.46	178.54 ± 4.35	156.33 ± 6.21
Triglyceride (mg/dl)	153.56 ± 12.64	127.49 ± 7.33	119.41 ± 8.33
HDL (mg/dl)	37.52 ± 1.61	43.00 ± 1.58	40.63 ± 1.44
LDL (mg/dl)	110.48 ± 9.99	111.06 ± 3.88	93.92 ± 4.31
tPA injection related results			
Time duration since symptom onset (hour)	102.48 ± 9.25	103.99 ± 5.24	92.61 ± 6.44
Door to needle time (hour)	67.12 ± 5.00	52.09 ± 3.73	61.98 ± 5.16
NIHSS after 36 h	3.03 ± 2.40	6.48 ± 5.124	17.89 ± 9.20
NIHSS after 7 days	2.34 ± 2.75	5.46 ± 5.72	15.28 ± 8.88
MRS after 7 days	1.45 ± 1.30	2.11 ± 1.40	4.01 ± 1.45

The patients were evaluated 7 days after the time of admission; however, the data was available only for 268 patients. The mean NIHSS was 2.7 ± 1.7 seven days after anti-thrombolytic therapy. The mRS was 3.14 ± 2.22 thirty-six hours after the treatment, which improved by 2.7 ± 1.73 in 7 days.

### Classification of results based on the RDW values

The patients’ characteristics according to the baseline RDW level is demonstrated in Table 2.

**Table 2:** Patients’ characteristics based on the baseline RDW level

	All the patients	RDW > 13%	RDW ≪ 12.9%
Age	64.89 ± 12.7	66.10 ± 11.8	61.28 ± 14.4
Length of stay	15.10 ± 16.4	15.08 ± 15.9	14.34 ± 18.5
Blood pressure (systolic)	154.52 ± 28.6	156.60 ± 28.80	148.58 ± 27.6
Blood pressure (diastolic)	89.63 ± 16.9	90.79 ± 17.7	86.33 ± 14.0
Body temperature	36.87 ± 0.2	36.88 ± 0.2	36.85 ± 0.2
Ejection Fraction	49.80 ± 8.3	49.45 ± 8.4	50.74 ± 8.1
White blood cell	8.3*10^3^	8.5*10^3^	7.7*10^3^
Hematocrit	41.39 ± 4.8	41.57 ± 5.1	40.88 ± 4.0
Platelet count	243*10^3^	239*10^3^	255*10^3^
INR	1.14 ± 0.8	1.17 ± 0.9	1.06 ± 0.1
PT	13.28 ± 1.2	13.33 ± 1.3	13.15 ± 0.8
PTT	28.70 ± 5.1	28.73 ± 4.7	28.60 ± 6.1
Creatinine	1.01 ± 0.3	1.02 ± 0.4	0.97 ± 0.1
Total cholesterol	170.19 ± 42.0	168.83 ± 40.8	174.33 ± 45.4
Triglyceride	125.43 ± 59.87	121.81 ± 58.9	136.51 ± 61.7
HDL	41.42 ± 11.1	41.20 ± 9.8	42.10 ± 14.4
LDL	105.33 ± 36.4	105.21 ± 34.1	105.69 ± 42.9

#### NIHSS

The categorization of RDW to ≪ 12.9% and > 13% values revealed insignificant difference in stroke severity score, accounting for the mean baseline NIHSS of 11.74 ± 6.39 in normal RDW values and 13.38 ± 0.49 in higher RDW group (p = 0.60). Similarly, the mean NIHSS of subjects with RDW < 12.9% was lower than the patients with RDW > 13% in each 36-h and 7-day evaluation, while the difference between two groups was statistically insignificant. The mean NIHSS was 8.19 ± 8.2 and 9.94 ± 8.28 in patients with normal and higher RDW values, respectively (p = 0.64). After 7 days, NIHSS was 6.46 ± 7.28 in normal RDW group and was 8.52 ± 8.35 in increased RDW group (p = 0.058).

#### mRS

The categorization of final outcome according to RDW level demonstrated mean mRS of 2.74 ± 1.56 within 36 h of tPA injection in the group of patients with normal RDW value, which was 3.25 ± 2.55 in increased RDW group. The final outcome results had a trend toward improvement in both RDW categories after 7 days. The mean mRS was 2.33 ± 1.59 and 2.72 ± 1.75 in normal and increased RDW group, respectively.

Neither the 36-h, nor the seventh day mRS demonstrated significant difference between those with normal and higher RDW values.

#### Length of stay at hospital

The length of stay at hospital in patients with RDW ≪ 12.9% was 14.34 ± 18.5 and in those with RDW > 13% was 15.08 ± 15.9. The results didn’t differ significantly between two groups (p = 0.96).

#### Symptomatic intracerebral hemorrhage

Symptomatic intracerebral hemorrhage (SICH) occurred in 14 patients, in 6 patients severe symptoms led to diagnosis and in 8 of them hemorrhage was asymptomatic. In patients with normal RDW level, 2.04% had symptomatic hemorrhage and 2.04% had asymptomatic hemorrhage. Among patients with elevated RDW, asymptomatic and symptomatic hemorrhage occurred in 4.69% and 3.35% of the patients. The analysis with Pearson’s test did not reveal a correlation between SICH and RDW.

### Three-month follow up

Out of 282 enrolled subjects, only 208 referred for the 3-month follow up session, 98 of them had a good final outcome with mean baseline RDW of 13.57 ± 1.35% and 110 had poor outcome on mRS evaluation with RDW of 13.76 ± 1.06%. The linear regression analysis didn’t address any significant regression between final outcome results and baseline RDW values (r = 0.04, p = 0.52).

**Fig. 1:**
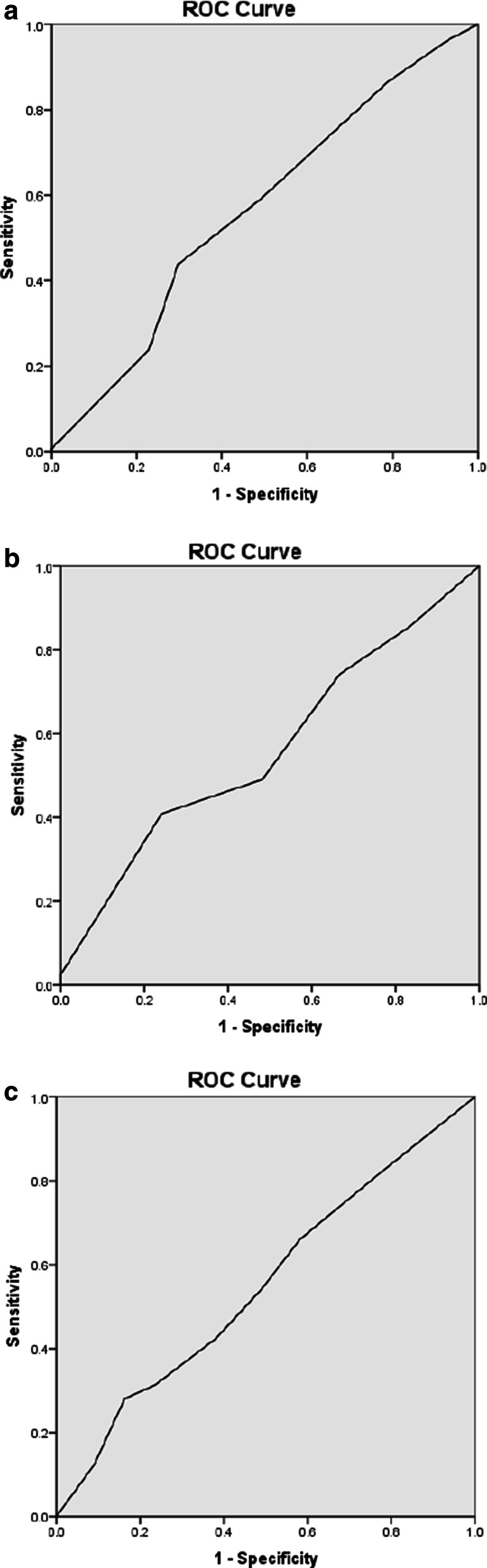
The ROC curve for outcome prediction of RDW in patients with ischemic stroke. **a** 36-h outcome prediction, **b** 7-day outcome prediction, **c** 3-month outcome prediction

The sensitivity, specificity and AUC of baseline RDW for predicting final outcome within 36 h were 77.6%, 69.8% and 0.51, respectively (Fig. 1a). In 7-day follow up, the sensitivity, specificity and AUC of baseline RDW were 75.0%, 74.3%, and 0.48, respectively (Fig. 1b). And during the period of 3 months the sensitivity, specificity and AUC of baseline RDW for mRS prediction were 74.4%, 71.4% and 0.57, respectively (Fig. 1c).

### Corrections for confounding factors

Multivariant linear regression analysis revealed only a significant correlation between age and stroke severity (p = 0.01) and outcome (p = 0.03). However, after corrections for the age there was still an insignificant relation between RDW and stroke severity and outcome (p = 0.20).

## Discussion

In this prospective cohort study, the association between RDW and stroke severity and patients’ final outcomes was assessed to identify the possible predictive value of RDW in patients with stroke who were administered antithrombotic therapy. The patients with higher RDW values at admission had more severe symptoms. Stroke severity improved after tPA administration in all patients. However, post-injection NIHSS did not differ significantly among those with high and low RDW values. Thus, there was not a significant correlation between stroke severity and RDW values in patients after tPA administration. In addition, the outcome scale in a 3-month interval follow up was not correlated with the patients’ baseline RDW values. Therefore, RDW from an approximate AUC of 0.5 with a cut-off score of 13 did not appear to be predictive of final stroke outcome.

In contrast with these findings, Kara et al. compared the RDW values in acute ischemic stroke patients in clusters with different severity scores and found RDW to be a predictive measure of stroke severity [15]. The authors also reported a significant correlation between RDW and other parameters, such as NIHSS and Glasgow comma score (GCS), and found RDW with a cutoff point of 14-which was higher than the current study- with higher sensitivity to differentiate stroke patients from normal subjects (AUC:0.76). In addition, Jia et al. studied 432 patients diagnosed with acute ischemic stroke and confirmed that RDW is closely related to the occurrence of ischemic stroke, revealing the importance of RDW in the progression of an ischemic stroke that may be related to carotid artery occlusion caused by large red blood cells [21].

In consistence, a population-based cohort study by Soderholm et al. [17] found high RDW values to associate with an increased risk of ischemic stroke.

While most of the previous findings are suggestive of the predictive value of RDW, Lappegård et al. reported that elevated RDW levels cannot predict the risk of stroke-induced mortality after exclusion for anemia [22]. However, Turcato et al. indicated that RDW could be used as an independent predictor of stroke severity and prognosis in patients with acute ischemic stroke who underwent antithrombotic therapy [23].

RDW is elevated in patients with ineffective erythropoiesis and may be associate with erythrocyte destruction. However, whether RDW is able to predict the incidence of stroke remains unclear. A population-based study with a normal control group might answer this question. In the present study, RDW did not predict the severity of stroke and final outcomes in those who underwent tPA injection. However, current study only included the patients with an acute ischemic attack and excluded other types of stroke, severe cases (those with NIHSS > 22), and those who were admitted later than 4.5 h after symptom onset. Therefore, the current study targeted more narrowed range of patients. Some studies have also proposed ischemia induced inflammation as a possible mechanism of increased RDW during stroke [14, 24]. However, the results of this study did not confirm the association between RDW and ischemia or restored blood flow. Further investigations with larger sample size are required.

## Limitations

Our study had some limitations to be considered. The population of our study included some patients with other medical conditions, which may have a confounding impact on findings. It is better to add that with regards to the pathologic nature of ischemic stroke which target aged population, the exclusion of such comorbities was impossible. Moreover, only the patients with ischemic stroke who were admitted within sufficient time for tPA injection were included in the study. So, the results cannot be generalized to all forms of stroke patients or with admission severity score. However, the study population in this study were followed prospectively and any possible factor that could affect the outcome measure were considered. Thus, the results are sufficient enough to suggest RDW does not predict the severity and outcome of ischemic stroke in patients who undergo antithrombotic therapy.

## Data Availability

The datasets used and analyzed during the current study are available from the corresponding author on reasonable request.

## Abbreviations

EDTA: Ethylenediaminetetraacetic acid
mRS: Modified Rankin Score
NIHSS: National Institute of Health Stroke Scale
RDW: Red blood cell distribution width
SICH: Symptomatic intracerebral hemorrhage
tPA: Tissue plasminogen activator
